# Novel components in the nuclear factor-kappa B (NF-κB) signaling pathways of endothelial cells under hyperglycemic-ischemic conditions

**DOI:** 10.3389/fcvm.2024.1345421

**Published:** 2024-05-24

**Authors:** Madhu V. Singh, Thomas Wong, Sonia Moorjani, Arul M. Mani, Ayotunde O. Dokun

**Affiliations:** Division of Endocrinology and Metabolism, Carver College of Medicine, University of Iowa, Iowa City, IA, United States

**Keywords:** endothelial cells, hyperglycemia, BLNK/BTK, peripheral artery disease, NF-κB, inflammation, diabetes, protein kinase C

## Abstract

Diabetes worsens the outcomes of a number of vascular disorders including peripheral arterial disease (PAD) at least in part through induction of chronic inflammation. However, in experimental PAD, recovery requires the nuclear factor-kappa B (NF-κB) activation. Previously we showed that individually, both ischemia and high glucose activate the canonical and non-canonical arms of the NF-κB pathway, but prolonged high glucose exposure specifically impairs ischemia-induced activation of the canonical NF-κB pathway through activation of protein kinase C beta (PKCβ). Although a cascade of phosphorylation events propels the NF-κB signaling, little is known about the impact of hyperglycemia on the canonical and non-canonical NF-κB pathway signaling. Moreover, signal upstream of PKCβ that lead to its activation in endothelial cells during hyperglycemia exposure have not been well defined. In this study, we used endothelial cells exposed to hyperglycemia and ischemia (HGI) and an array of approximately 250 antibodies to approximately 100 proteins and their phosphorylated forms to identify the NF-κB signaling pathway that is altered in ischemic EC that has been exposed to high glucose condition. Comparison of signals from hyperglycemic and ischemic cell lysates yielded a number of proteins whose phosphorylation was either increased or decreased under HGI conditions. Pathway analyses using bioinformatics tools implicated BLNK/BTK known for B cell antigen receptor (BCR)-coupled signaling. Inhibition of BLNK/BTK in endothelial cells by a specific pharmacological inhibitor terreic acid attenuated PKC activation and restored the IκBα degradation suggesting that these molecules play a critical role in hyperglycemic attenuation of the canonical NF-κB pathway. Thus, we have identified a potentially new component of the NF-κB pathway upstream of PKC in endothelial cells that contributes to the poor post ischemic adaptation during hyperglycemia.

## Introduction

Diabetes significantly worsens the outcomes of a number of vascular disorders including peripheral arterial disease (PAD) (1–6). This poor outcome is attributed in part to the inflammatory milieu of the diabetic condition (1, 7–10). The NF-κB signaling pathway is one of the most studied inflammatory pathways under multiple stress and disease related conditions including diabetes (11–13). The NF-κB transcription factor is a heterogeneous group of five transcription factor subunits that form dimers in various combinations to regulate transcription of their various target genes (14, 15).

The commonly described NF-κB signaling pathway, also known as the canonical pathway, involves phosphorylation dependent tagging and subsequent proteosomal degradation of the inhibitory IκBα subunit to allow nuclear translocation of the p65 subunit containing NF-κB dimer (14). NF-κB signaling is activated under ischemic conditions and is required for arteriogenesis following an ischemic injury (16). Inhibition of NF-κB signaling pathway in endothelial cells shows aberrant endothelial function and poor post-ischemic perfusion recovery (16). Recently, we have shown that chronic activation of NF-κB pathway by hyperglycemia in diabetes impairs activation of the canonical NF-κB pathway as measured by increased IκBα levels (1). Moreover, we showed that, this process involves activated PKCβ and contributes to poor reperfusion recovery in a preclinical model of peripheral artery disease (PAD) in type I diabetes (1). Inhibition of PKCβ by ruboxistaurin (Rbx) restored the canonical NF-κB signaling pathway both *in vitro* and *in vivo* suggesting a pivotal role of PKCβ activity in post ischemic adaptation in the setting of hyperglycemia. However, the molecular signals and pathways that lead to PKCβ activation under hyperglycemic ischemic conditions are not well understood. To elucidate the pathway involved, we used a combined approach of bioinformatics and unbiased screening of the phosphorylation states of approximately 100 proteins known to participate in various pathways of NF-κB activation. Here we report a previously unknown signaling pathway of NF-κB activation in endothelial cells that resembles B cell receptor signaling.

## Materials and methods

### Cell cultures

All experiments were performed using pooled human vascular endothelial cells (HUVEC, ATCC, Cat # PCS-100-011, USA) and human aortic endothelial cells (HAEC) from Cell Applications (Cat # 304-05a, San Diego, CA, USA) or mouse microvascular endothelial cells from skeletal muscles (MMEC, Cat#T4991, Applied Biological Materials, Richmond, British Columbia, Canada) between passage 4 and 7 as described previously (1). The complete endothelial cell growth medium (ECGM) was obtained from Cell Applications, Inc. (Cat # 211-500, San Diego, CA, USA). All endothelial cells were grown on gelatin coated (Cat # 6950, Cell Biologics) plastic culture dishes in the above medium under 95% humidity and 20% O_2_. For simulated ischemia, culture medium was removed, cells were washed with Dulbecco's PBS and medium was replaced with endothelial cell starvation medium (Cell Applications, San Diego, Cat# 209–250) and incubated for 24 h in a hypoxia chamber with 95% humidity and 2% O_2_ (6). Cell cultures were maintained in normal glucose (5 mM D-glucose). For experiments, the medium was supplemented with 20 mM D-glucose (high glucose medium, HG) or with 20 mM metabolically inert L-glucose (normal glucose medium, NG). For inhibitor studies, cultured cells were treated with terreic acid at described concentrations shown in results (Cat# 1405, Tocris). For BLNK knockdown by shRNA, MMEC were transfected with plasmid containing shRNA sequence (TRCN0000329213, Millipore-Sigma) or corresponding empty plasmid vector using Lipofectamine3000 (Thermo-Fisher). Cell lysates were prepared in RIPA buffer and immunoblotting performed.

### Phosphoprotein array

Antibodies arrays were used to detect differences in phosphorylation of signaling molecules involved in the NF-κB pathway (NF-κB Phospho Antibody Array, Cat# PNK215, Full Moon Biosystems, Sunnyvale, CA, USA) as described previously (1). These arrays contained 215 antibodies to approximately 100 proteins and their phosphorylated forms that are known to participate in the NF-κB signaling pathways. Cell extracts from HUVEC exposed to either normal glucose (NG, 5 mM D-Glucose), normal glucose with ischemia (NGI), 25 mM D-glucose (HG), or 25 mM D-glucose with ischemia (HGI) were analyzed for the phosphoprotein levels according to the manufacturer's suggested protocol. A microarray scanner was used to record and digitize the fluorescence signals. Raw data were analyzed by manufacturer's recommendations using ImageJ (17) and transferred to Excel worksheet (Microsoft Office suite) for further analyses. Background corrections were performed, and the signals normalized against the median intensity of all the experimental spots on the array. Fold change in protein phosphorylation was calculated by dividing the intensity of the phosphorylated spot by the signal intensity of the corresponding non-phosphorylated spot for each protein. Differential expression between NG and NGI samples, and HG and HGI were calculated by dividing the phosphorylation ratio of the NGI and HGI with that of the NG and HG controls, respectively. Significant change was taken as greater than 1.5- or less than 0.67-fold. This experiment was carried out with three samples for each experimental group, and the array contained six spots for each antibody.

### Immunoblotting

Cell and tissue lysates were prepared in RIPA buffer (Thermo Scientific, Cat# 89901) as described previously (1). Equal amounts of protein in each sample were separated on NuPAGE gels and transferred to nitrocellulose membranes (BioRad, Cat# 1620094). After blocking non-specific binding in Intercept blocking buffer (LiCor, Cat# 927-60001), the membranes were probed with primary antibodies overnight at 4°C. The primary antibodies used were anti-IκBα (Cat#9242, Cell Signaling Technology, Danvers, MA, USA), anti-pSer661-PKC (Cat# DBOA15543, Vita Scientific, Beltsville, Maryland), anti-BTK (Cat# 8547, Cell Signaling Technology, Danvers, MA, USA), anti-BLNK (Cat# 36438, Cell Signaling), phospho-BLNK-Tyr84 (Cat # 26528, Cell Signaling), anti-GAPDH (Cat# 2118), and anti-β-actin (Cat# 3700, Cell Signaling). Following washes with Tris-buffered saline-0.1% Tween20 (TBST), secondary antibodies either Donkey-anti-rabbit-HRP (Cat # NA93AV, Cytiva), Goat-anti-rabbit-IR800, or Goat-anti-mouse-IR680 (both Li-COR Biosciences, Lincoln NE, USA) were used at 1:5,000 dilution in blocking buffer for 1 h at room temperature. Membranes were washed in TBST and signals were captured by iBright 1500 (Invitrogen) imager either directly for IR antibodies or by enhanced chemiluminescence (ECL) method. Quantification of the protein bands on immunoblots were performed using Image Studio Lite version 5.2 (Li-COR Biotechnology, Lincoln, Nebraska). Abundance of phosphoproteins was determined as the ratio of band intensity of target protein bands to β-actin.

### RNA isolation and real-time QPCR

RNA was isolated from cultured cells using Direct-zol RNA mini prep kit (Zymo Research, USA, Cat. R2052)) using Tri Reagent for cell lysis. Quality of isolated RNA (A260/280 ≥2.0) and quantification was done on a NanoDrop instrument. Aliquots of 200 ng RNA samples were reverse transcribed using a High-Capacity RNA to cDNA kit (AppliedBiosystems, Cat 4388950). For QPCR of BTK RNA, 10 ng RNA equivalent cDNA was used in each reaction with Power SYBR Green reagent (AppliedBiosystems, Cat 4367659) on a QuantStudio 3 thermocycler (ThermoFisher). One picomoles of forward and reverse primers were used for *BTK* (Forward 5′-AGCACAACTCTGCAGGACTC-3′ and Reverse 5′- TGCAGTGGAAGGTGCATTCT-3′) and *GAPDH* (Forward 5′- GTCTCCTCTGACTTCAACAGCG-3′ and Reverse 5′-ACCACCCTGTTG CTGTAGCCAC-3′). *GAPDH* was used as a loading control (1 ng RNA equivalent for GAPDH QPCR) and expression analysis was done by ΔΔCt method (18).

### Statistical analysis

The measurements are expressed as mean ­± SEM. Statistical comparisons between two groups (e.g., treated vs. untreated) were performed by *t*-test, whereas for more than two groups, we used one-way analysis of variance. A *P* value of <0.05 was considered statistically significant.

## Results

In hyperglycemic mice with type 1 diabetes, perfusion recovery and post ischemic adaption is poor following experimental PAD or induction of hind limb ischemia (HLI) (1, 19, 20). We have shown that ischemic activation of the canonical NF-κB pathway, is impaired under hyperglycemic conditions (1). Therefore, we investigated endothelial cells for an increase or decrease in phosphorylation of proteins associated with NF-κB signaling under hyperglycemic-ischemic conditions. Using HUVECs grown either in normal glucose with ischemia (NGI) or high D-glucose with ischemia (HGI), we compared the phosphorylation state of 98 protein sites representing about 48 distinct proteins. These proteins are known to be modulated in various pathways of the NF-κB signaling. We used an array of antibodies and selected the protein phosphorylation sites whose signals were increased greater than 1.5-folds or decreased greater than 0.67-fold change.

**Figure 1:**
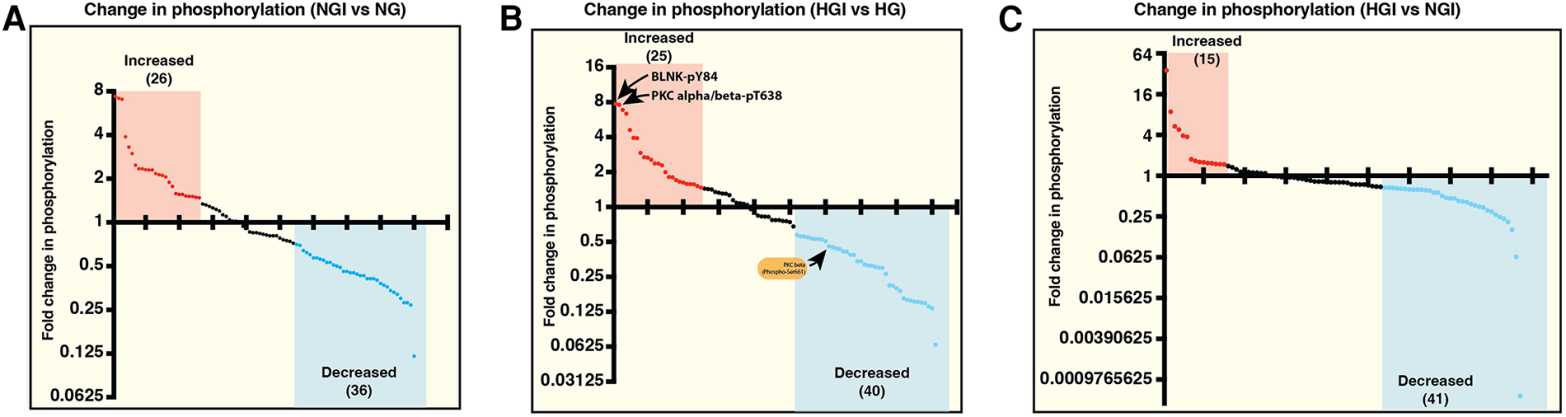
Measurement of relative changes in the phosphorylation state of proteins involved in the signaling pathways of NF-κB activation using printed arrays of antibodies. The graphs show fold-changes as ratios of phosphorylated/total signal in human endothelial cell lysates. (**A**) The plot shows fold-change in phosphorylation of specific epitopes of proteins in human endothelial cells under normal D-glucose and ischemic condition (5 mM D-glucose + 20 mM L-glucose, NGI) compared to the control cells grown under normal D-glucose and normoxic condition (5 mM D-glucose + 20 mM L-glucose, NG). Each dot represents a phospho-protein site. Red and blue dots represent greater than 1.5-folds increase or greater than 0.67-fold decreased phosphorylation, respectively. (**B**) Comparison of changes in protein phosphorylation in hyperglycemic-ischemic endothelial cells. The plot shows protein sites with 1.5-folds increased (red dots) or 0.67-fold decreased (blue dots) phosphorylation in high D-glucose and ischemia (25 mM D-glucose + ischemia, HGI) compared to high D-glucose and normoxia (25 mM D-glucose, HG). (**C**) Phospho-proteins comparison of NGI and HGI (1.5-folds increase or 0.67-fold decrease cutoff) revealed increased phosphorylation at 15 sites (red dots) and decreased phosphorylation at 41 sites (blue dots). *N* = 3 antibody arrays per group, 1 array/sample.

We first analyzed the increase or decrease in phosphorylation of signaling proteins in cells grown in normal glucose concentration and exposed to either normoxia (NG) or ischemia (NGI). In NGI samples, when compared to normoxic samples (NG), 26 protein phosphorylation sites were identified to undergo increased phosphorylation whereas 36 sites had decreased phosphorylation (Figure 1A). Similarly, when cell cultured in high D-glucose (HG) and exposed to normoxia were compared to cells exposed to ischemia (HGI) increased phosphorylation of 25 protein sites and decreased phosphorylation of 40 sites (Figure 1B) were identified. Additionally, a direct comparison of NGI and HGI results showed increased phosphorylation on 15 sites and decreased phosphorylation on 41 sites (Figure 1C, also see Supplementary Material Table S1). Thus, ischemia modulates phosphorylation of a different number of sites in proteins that are involved in NF-κB signaling pathway under normal glucose compared to high glucose.

**Figure 2:**
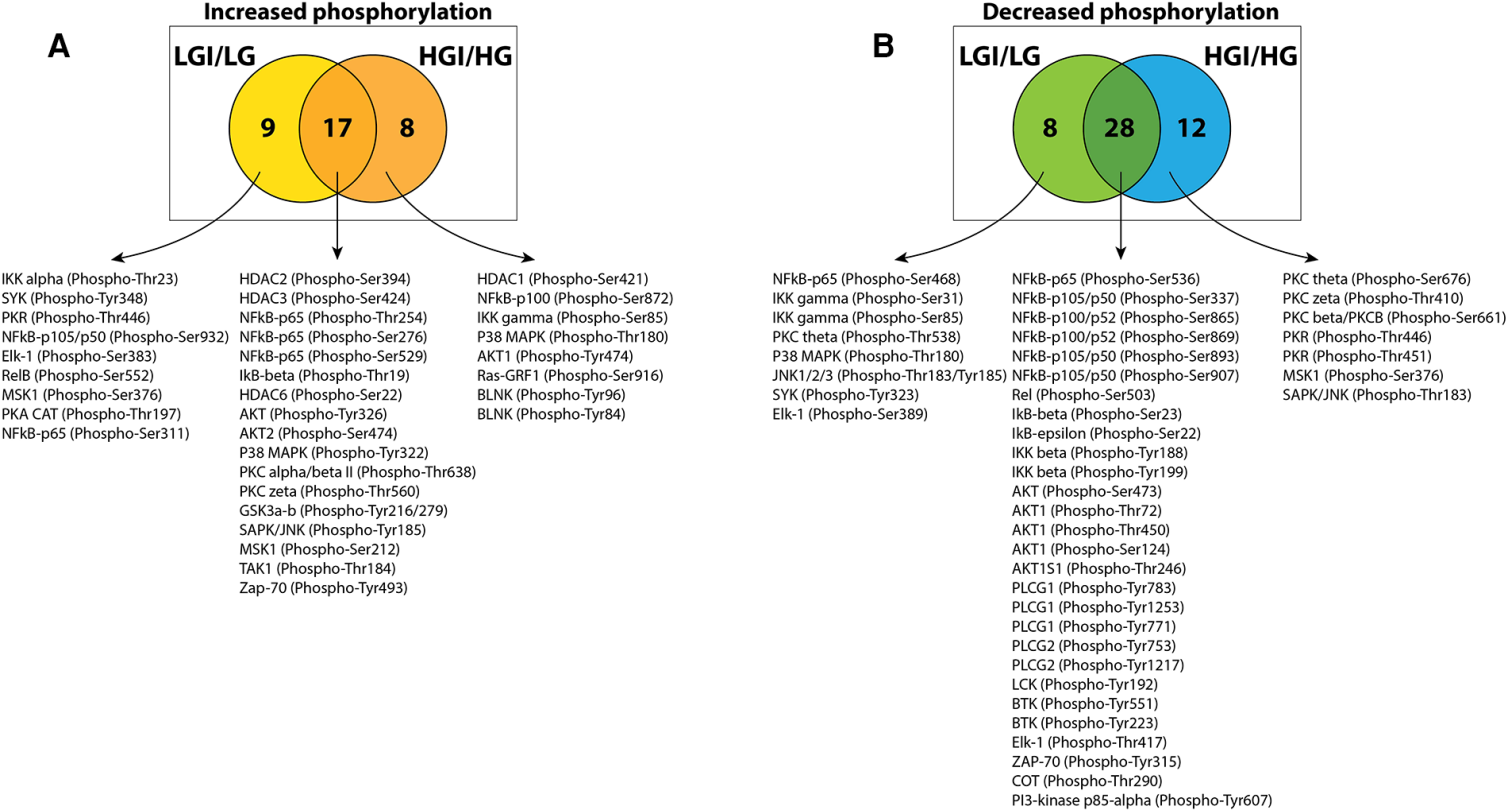
Venn diagram analysis of protein phosphorylation sites with 1.5-folds changes in normal D-glucose with ischemia (NGI) and high D-glucose with ischemia (HGI) to identify specific protein phosphorylation sites in hyperglycemic-ischemic conditions. (**A**) Comparison of the protein sites with increased phosphorylation in NGI and ischemic HGI showed 9 NGI-specific and 8 HGI-specific protein phosphorylation sites. (**B**) Comparison of sites with decreased phosphorylation showed 8 NGI-specific sites and 12 HGI-specific sites.

To identify protein phosphorylation in ischemic ECs that could be attributed to exposure to high glucose we compared the list of sites that had increased phosphorylation in either NGI or HGI. A Venn-diagram analysis showed that out of the 25 sites whose phosphorylation was increased in HGI, 17 sites were common to both NGI and HGI, suggesting that these increases were related to ischemia, independent of glucose levels (Figure 2A). There were 9 out of 26 sites that had increased phosphorylation specifically in NGI, suggesting ischemia induced changes under normal glucose conditions. In addition, 8 sites had increased phosphorylation only in HGI samples indicating their unique role in hyperglycemic ischemia.

Similarly, comparison of the list of sites with decreased phosphorylation in either NGI or HGI showed that 28 sites had decreased phosphorylation under ischemic conditions irrespective of the glucose concentration in the growth medium. There were 8 sites with decreased phosphorylation only in NGI and 12 sites whose phosphorylation decreased only in HGI (Figure 2B).

**Figure 3:**
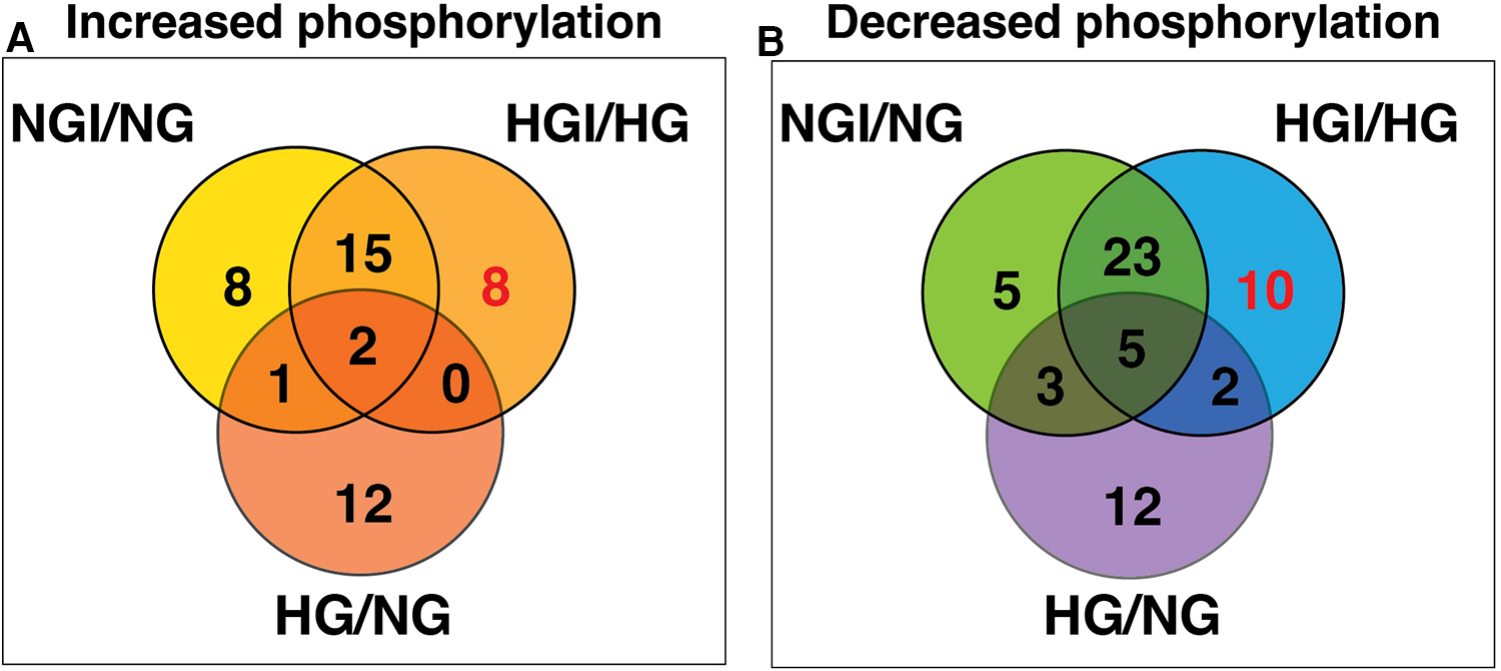
Venn diagram analyses to impose a higher level of specificity of phosphorylation changes by eliminating high D-glucose-related phosphorylation sites (HG). The 15 HG-related increased and 22 decreased phosphorylation sites were obtained from our published results (1). These analyses eliminated sites that whose phosphorylation is either increased (**A**) or decreased (**B**) in the presence of high D-glucose alone. The analysis yielded 8 HGI-specific sites with increased phosphorylation and 10 HGI-specific sites that had decreased phosphorylation (shown in red).

Since high D-glucose alone can induce NF-κB signaling, we further compared these increases or decrease in protein phosphorylation with the respective protein sites observed in high D-glucose-specific conditions (NG/HG) (Figure 3A). There was increased phosphorylation of 15 protein sites attributable to HG, 3 of these sites showed increased phosphorylation also in ischemic conditions with NG while 2 other sites showed increased phosphorylation in HGI. Of the 8 phospho-sites identified in HGI/HG (Figure 2A), all were specific for high D-glucose with ischemia (HGI, Figure 3A). However, of the 12 decreased phospho-proteins in HGI (Figure 2B), 10 protein sites were specific for high D-glucose with ischemia, whereas 2 protein sites also had decreased phosphorylation in high D-glucose (HG/NG) without ischemia (Figure 3B). Thus, high glucose, ischemia, or combinations of these conditions result in specific changes at different phosphorylation sites of proteins in the NF-κB signaling pathways (Table 1).

**Table 1:** Hyperglycemia-ischemia (HGI) specific change in phosphorylation of signaling proteins involved in the NF-κB pathway.

Phospho-protein site	Change in phosphorylation	Reference to NF-κB involvement
P38 MAPK (Phospho-Thr180)	Up	(21–23)
AKT1 (Phospho-Tyr474)	Up	(24)
Ras-GRF1 (Phospho-Ser916)	Up	(25, 26)
HDAC1 (Phospho-Ser421)	Up	(27)
NF-κB-p100 (Phopho-Ser872)	Up	(28)
BLNK (Phopho-Tyr96)	Up	(29)
BLNK (Phopho-Tyr84)	Up	** **
IKK gamma (Phopho-Ser85)	UP	(30)
PKC theta (Phopho-Ser676)	Down	(31, 32)
PKR (Phospho-Thr446)	Down	(33)
PKR (Phopho-Thr451)	Down	** **
PKC beta (Phospho-Ser661)	Down	** **
PKC zeta (Phospho-Thr410)	Down	(31, 32, 34)
HDAC5 (Phopho-Ser259)	Down	** **
SAPK/JNK (Phospho-Thr183)	Down	** **
IκB-alpha (Phopho-Ser32/36)	Down	(35)
NF-κB-p65 (Phopho-Thr435)	Down	(36)
LCK (Phopho-Tyr505)	Down	** **

Multiple signaling pathways related to environmental stress affect development, growth, cell death and post-ischemic adaption through activation of the NF-κB pathway (37). To identify the pathway leading to NF-κB activation in hyperglycemic-ischemic condition in endothelial cells, we employed a bioinformatics approach. We hypothesized that any protein that has undergone a change in phosphorylation under HGI, irrespective of the direction of change, must participate in the NF-κB signaling pathway. Moreover, change in environmental condition may not necessarily recruit an entirely new signaling pathway; instead, modification of even a single key protein may potentially alter the signaling outcome. Therefore, to identify the signaling pathway involved in hyperglycemic-ischemic condition (HGI), we obtained a list of genes representing all phosphoproteins that were modulated in HGI (Figure 1B). The 65 modulated phospho-sites (25 increased and 40 decreased) in HGI (Figure 1B) were represented by 35 genes (Table 2). We then performed an over-representation test on this set of representative genes using PANTHER molecular classification system (38).

**Table 2:** The list of 35 distinct endothelial cell proteins within the NF-κB signaling pathway showing change in phosphorylation under hyperglycemic-ischemic conditions (HGI).

Proteins in HGI	Genes in HGI
AKT1	AKT1
AKT2	AKT2
BLNK	BLNK
BTK	BTK
CSNK2B	CSNK2B
PKR	EIF2AK2
ELK-1	ELK1
GSK3a	GSK3A
GSK3b	GSK3B
HDAC1	HDAC1
HDAC5	HDAC5
IκB beta	IKBKB
IKK gamma	IKBKG
LCK	LCK
TAK1	MAP3K7
COT	MAP3K8
P38 MAPK	MAPK14
SAPK/JNK	MAPK8
NF-κB p105/p50	NFKB1
NF-κB p100/p52	NFKB2
IκB-alpha	NFKBIA
IKK beta	IKBKB
IκB epsilon	NFKBIE
PI3-kinase p85 alpha	PIK3R1
PLCG1	PLCG1
PLCG2	PLCG2
PKC alpha	PRKCA
PKC beta II	PRKCB
PRKCQ	PRKCQ
PKC zeta	PRKCZ
RAS-GRF1	RASGRF1
REL	REL
NF-κB p65	RELA
MSK1	RPS6KA5
ZAP-70	ZAP70

**Figure 4:**
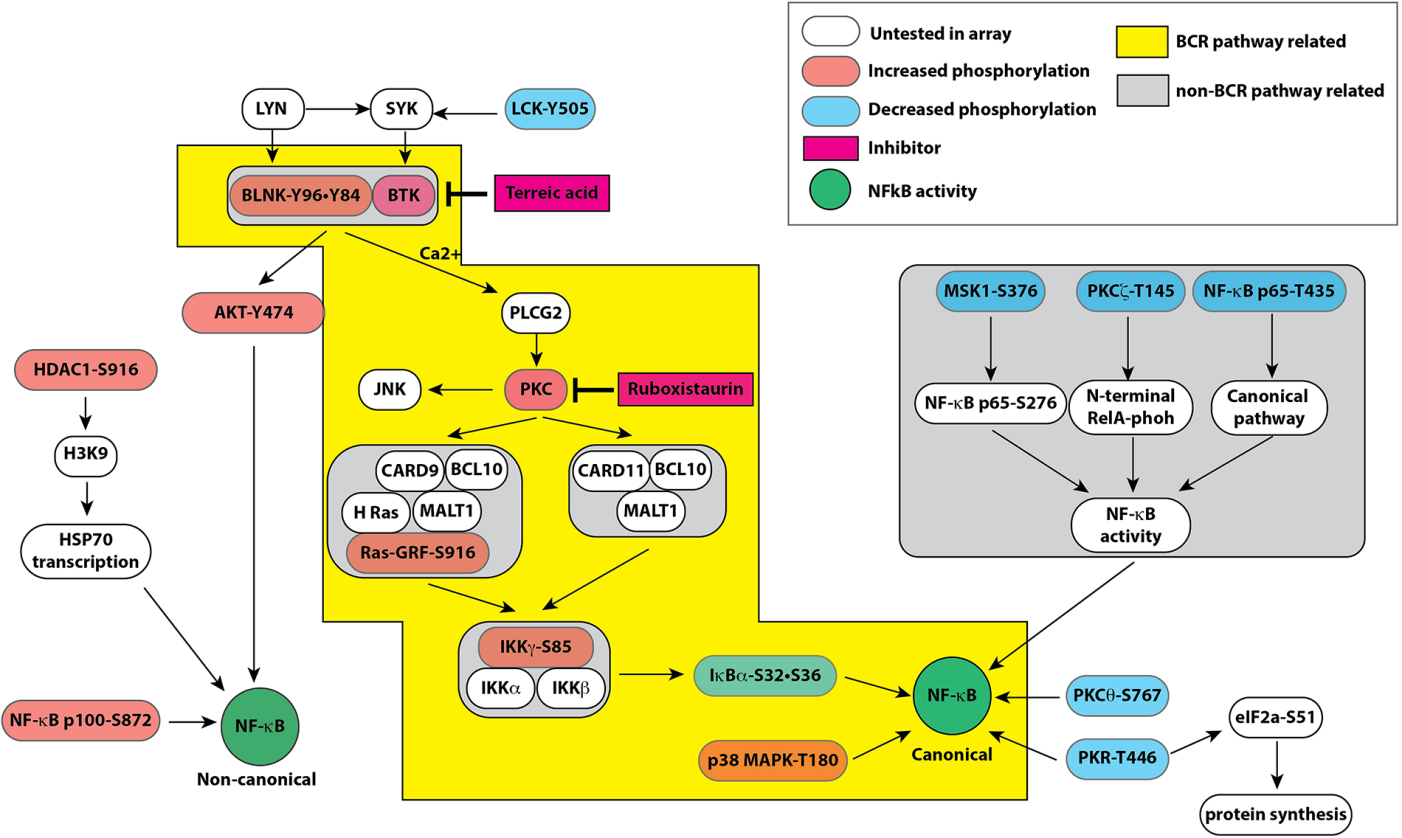
Pathway map based on combined results obtained from bioinformatic analyses of signaling protein phosphorylation sites using PANTHER molecular classification system and reactome database. Both databases strongly suggested a role of B cell receptor like pathway in endothelial cells leading to modulation of the canonical and non-canonical pathways of NF-κB activation by hyperglycemic-ischemic conditions. The colored boxes show proteins represented in the experimental data. The components highlighted in orange show increased phosphorylation whereas components highlighted in blue represent decreased phosphorylation on the depicted proteins. Please see the text for description.

Among the predicted pathways, the toll-like receptor pathway, B cell activation and T cell activation pathways were prominent for their fold-enrichment (TLR >100-folds, T cell activation 81.71-folds, and B-cell activation 80.56-folds) and low probability of false discovery rate (Table 3). Similar results were obtained from the Reactome database (www.reactome.org) for enrichment of genes related to the TLR and B cell receptor signaling (BCR) pathways. Together, these results suggest that in addition to the TLR pathway, the endothelial cells may also have an operative BCR like pathway (Figure 4). Although BCR pathway was originally thought to be limited to the B lymphocytes, it has now been known to operate in several non-B cells as well (39, 40). However, since this pathway or its components have not been reported in endothelial cells, we decided to investigate this pathway.

**Table 3:** Identified NF-κB signaling pathways based on PANTHER pathways over-representation (*homo sapiens*) analysis of proteins whose phosphorylation was modulated by hyperglcemic-ischemic (HGI) condition. Top 5 pathways are shown. PANTHER overrepresentation test (released 20240226; PANTHER version 18.0 (released 2023-08-01).

PANTHER pathway	Number of genes	Fold enrichment	P value	FDR (false discovery rate)
Toll receptor signaling pathway	12	>100	8.78E-23	7.07E-21
T cell activation	12	81.71	1.08E-20	4.35E-19
B cell activation	10	80.56	2.90E-17	7.79E-16
Insulin/IGF pathway-protein kinase B signaling cascade	5	75.26	5.89E-09	4.51E-08
Histamine H1 receptor mediated signaling pathway	6	74.61	1.64E-10	1.76E-09

### BLNK/BTK-dependent NF-κB signaling in endothelial cells

**Figure 5:**
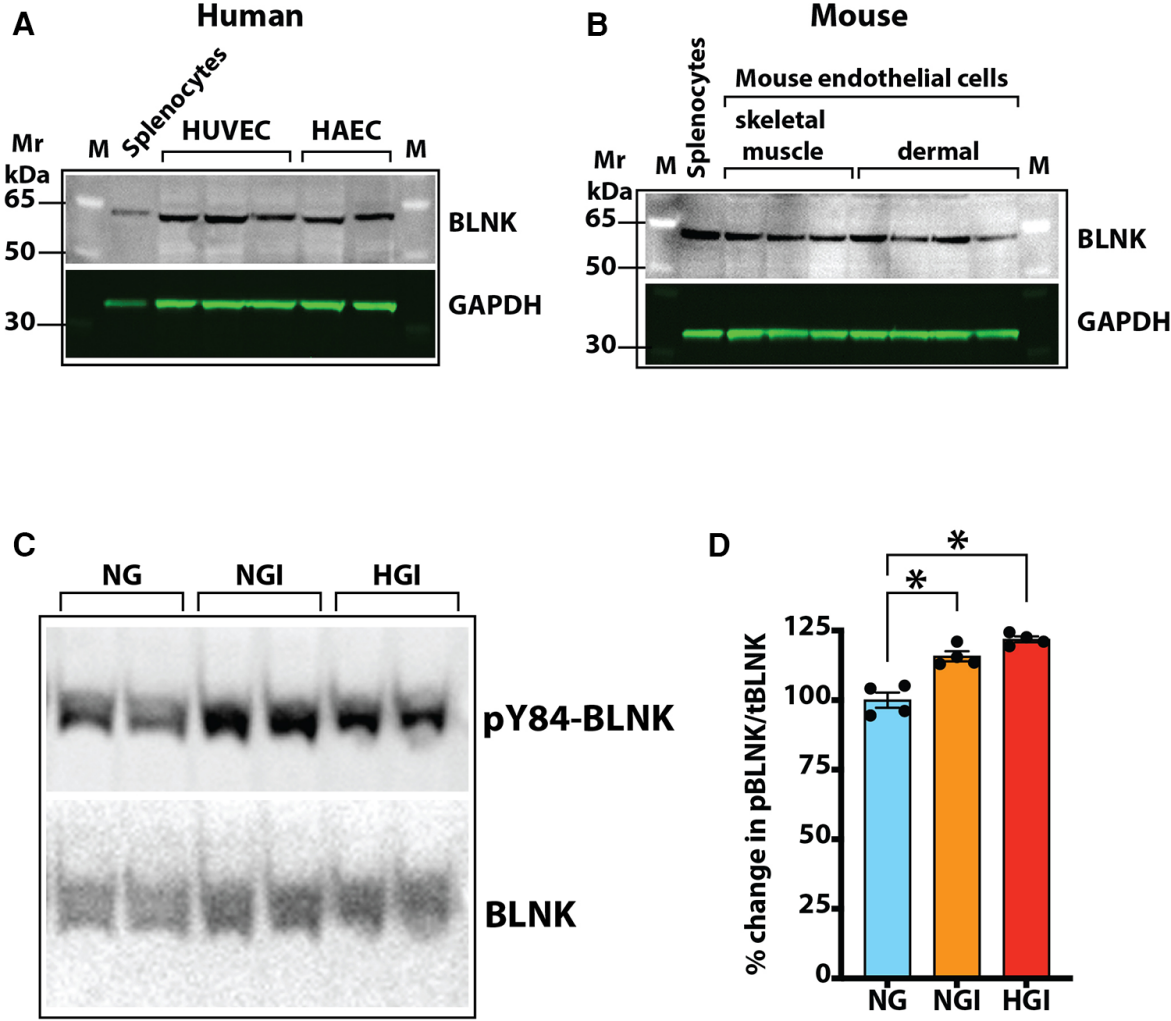
Expression of BLNK in endothelial cells. (**A**) Immunoblotting of primary cultures of human umbilical vein endothelial cells (HUVEC) and human aortic endothelial cells (HAEC) for BLNK protein. Each lane represents a replicate cell culture of the labeled primary cell type. (**B**) Immunoblotting of mouse primary endothelial cells of skeletal muscle or dermal origin for BLNK protein. GAPDH was used as a loading control in the lanes. Each lane represents a replicate culture of the respective primary cell type. (**C**) Immunoblots showing phosphorylated BLNK protein on Tyr84 residue (pY84-BLNK) under normal glucose plus ischemia (NGI) and high glucose plus ischemia (HGI) in mouse skeletal muscle endothelial cells, (**D**) pY84-BLNK in mouse skeletal muscle endothelial cells is increased under both NGI and HGI conditions (*n* = 4 each group, asterisks denote *p* < 0.05).

**Figure 6:**
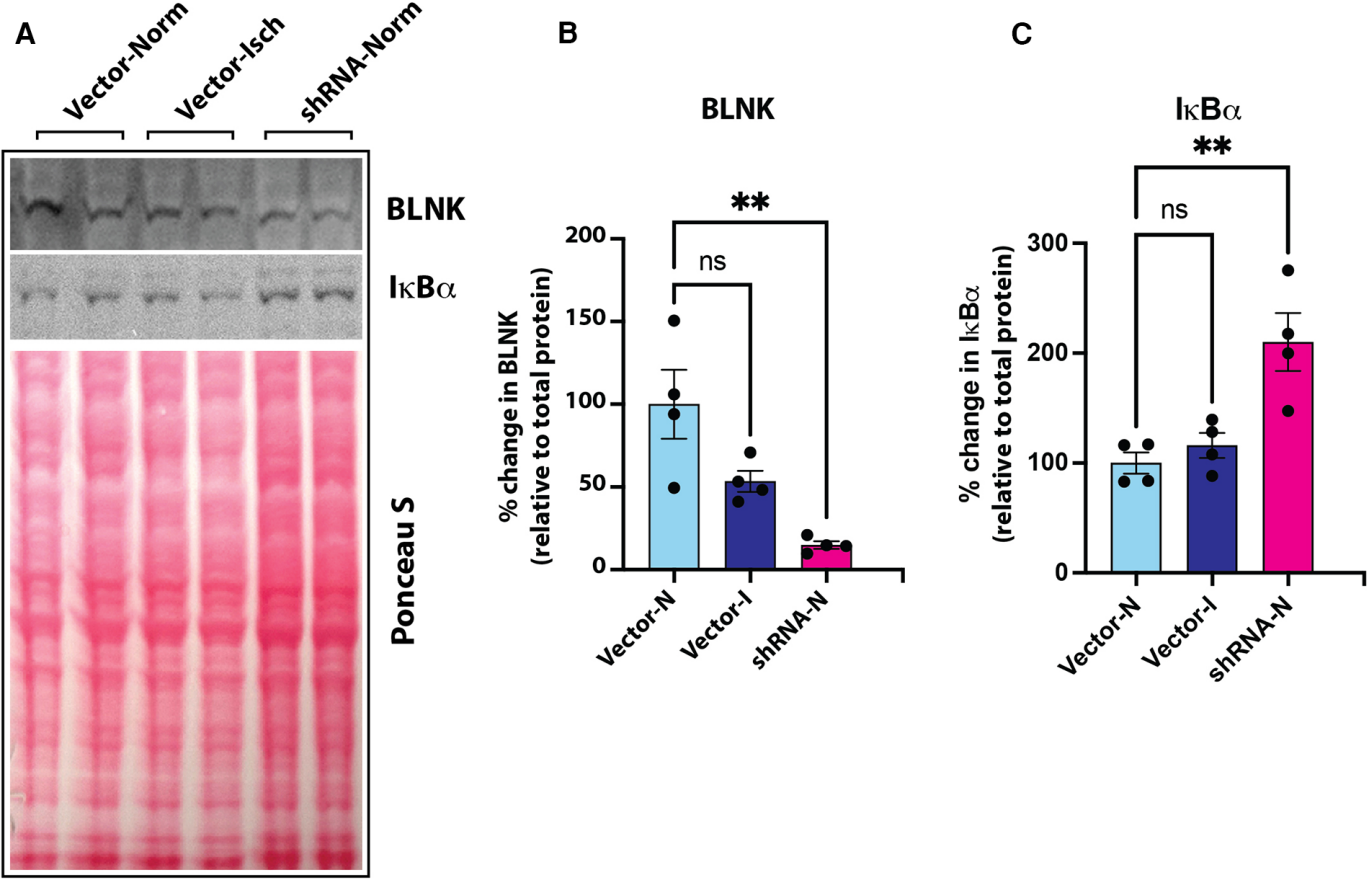
(**A**) Immunoblot of BLNK and IκBα proteins in culture grown primary mouse skeletal microvascular endothelial cells (MVECsk). Intensity of protein bands was normalized to total loaded protein in corresponding lanes (Ponceau S staining). (**B**) BLNK protein was significantly knocked down by transfection of shRNA harboring plasmid (shRNA-Norm) compared to control plasmid vector transfected cells and kept under normoxic or ischemic conditions (Vector-Norm and Vector-Isch, respectively). (**C**) Knockdown of BLNK resulted in increased IκBα protein in corresponding samples probed on the same blot. *N* = 4 each group, *p* < 0.05 by One Way ANOVA).

Our screening showed a role of B-cell linker protein (BLNK) in the predicted B-cell receptor activation pathway. BLNK is responsible for B-cell receptor (BCR) signaling pathway leading to B-cell development (41). BLNK is a scaffold protein that bridges BCR signaling components with downstream signaling pathways including activation of the NF-*κ*B signaling pathway (42). In addition, BLNK has also been shown to play a role in growth of cancer cells (39, 40). However, their presence and role in endothelial cell signaling is not known. We used antibody to BLNK in immunoblotting experiments with several primary endothelial cells from human and mouse. We found that BLNK protein is expressed in human (HUVEC, HAEC, Figure 5A) as well as mouse (MVECsk, MVEC) endothelial cells (Figure 5B). In addition, BLNK protein undergoes increased phosphorylation at the Tyr84 residue (pY84-BLNK) under ischemic conditions both in normal glucose (NGI) and high glucose (HGI) as predicted from the phosphoarray screening (Figures 5C,D). These results validated the results from the phosphoarray screening that BLNK protein is expressed in endothelial cells and its phosphorylation is modulated under ischemic conditions. To test the effect of BLNK on the NF-κB pathway, we used shRNA-mediated knockdown of BLNK protein in MVECsk cells (Figures 6A,B). Decrease in BLNK resulted in decreased NF-κB basal activity reflected by increase in steady state levels of IκBα protein (Figures 6A–C) suggesting that BLNK participates in the NF-κB signaling pathway in endothelial cells.

**Figure 7:**
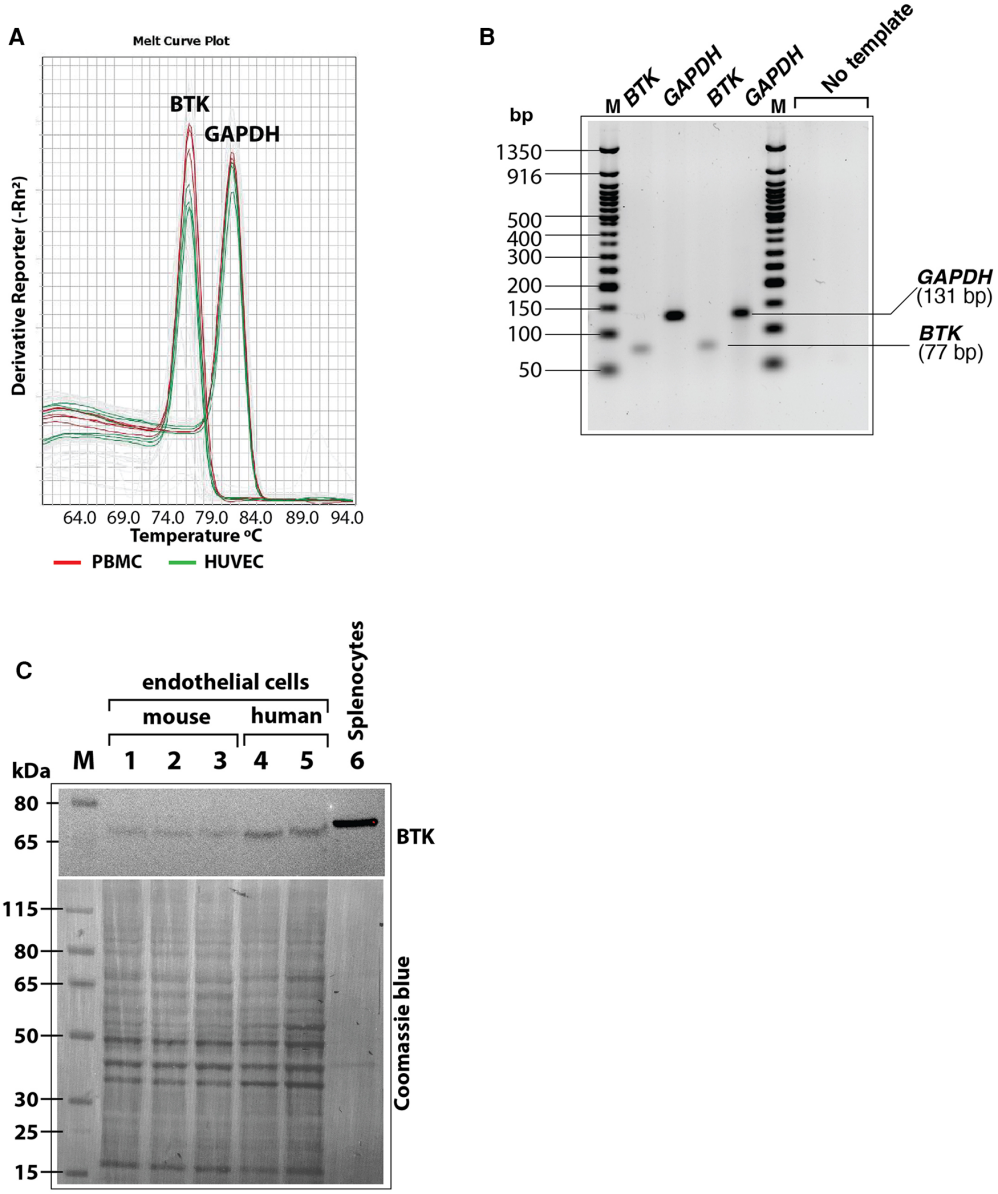
(**A**) Melt curves of BTK and GAPDH amplicons after RT-QPCR run showing similar *BTK* and *GAPDH* PCR product peaks from human peripheral blood mononuclear cells (PBMC, red lines) and HUVEC (green lines). (**B**) RT-QPCR reaction products were analyzed by agarose gel electrophoresis (3% agarose-TBE gel) to show the BTK amplicon (77 bp) and GAPDH amplicon (131 bp) in two different reactions were of specific and of expected size. Each lane represents samples loaded from an RT-QPCR reaction product. (**C**) Immunoblot of mouse and human primary endothelial cell lysates showing BTK protein expression. After probing with antibody, the blot was stained with Coomasie blue stain to visualize loading of protein bands in each lane. Less total protein was loaded in the mouse splenocytes lysate (positive control). Protein molecular size marker locations are shown on the left.

As a scaffold protein, BLNK recruits multiple proteins to propagate downstream signaling pathways including PLCγ activation (43). Bruton's tyrosine kinase (BTK) is a BLNK-associated non-receptor tyrosine kinase that is important for this signaling (44). Therefore, we tested the expression of BTK mRNA and protein in cultured human endothelial cells (HUVEC and HAEC) by RT-QPCR and immunoblotting, respectively. In RT-QPCR analysis using BTK-specific primers, the expression of BTK was detected both in HUVEC and peripheral blood mononuclear cells (PBMC) as seen by melt curve analysis of SYBR green based RT-QPCR products of BTK and GAPDH. Result showed similar peaks in PBMC and HUVECS with similar Tm values (Figure 7A). Agarose gel electrophoresis of the RT-QPCR reaction products showed single specific band of expected molecular size (77 bp) for BTK amplicon and 131 bp amplicon for GAPDH (Figure 7B). Immunoblotting of cell lysates also showed BTK protein expression in human as well as mouse cultured primary endothelial cells (Figure 7C). Together, these results strongly suggested that BLNK and BTK are expressed in HUVEC as well as other endothelial cells from human and mouse and validate the results of the phosphoarray screening.

**Figure 8:**
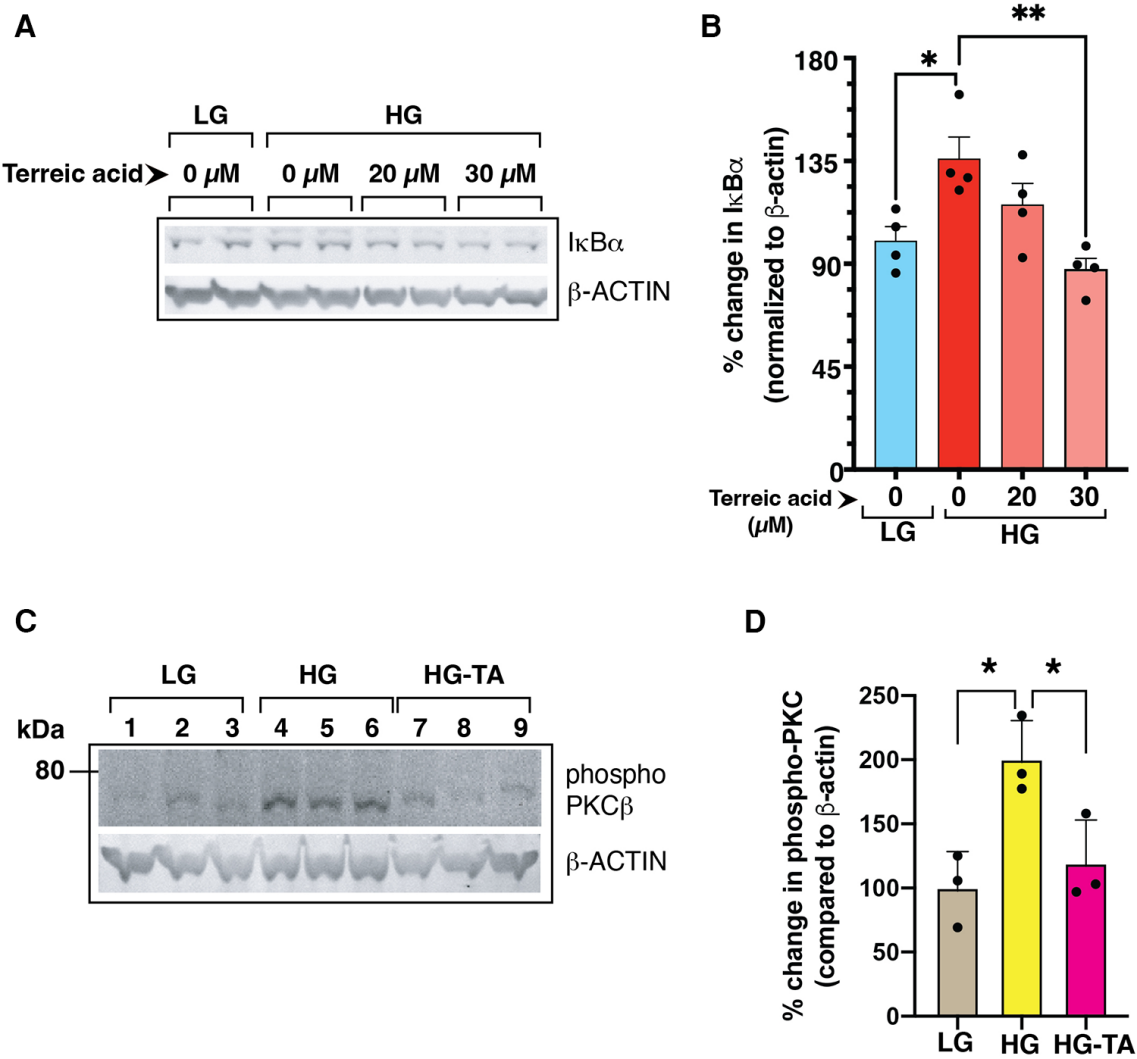
(**A**) Mouse primary endothelial cell cultures were subjected to normal glucose (NG, 5 mM D-glucose) or high D-glucose (HG, 25 mM D-glucose) exposure for 3 days in the presence or absence of a selective BTK inhibitor terreic acid (0, 20 and 30 µM final concentration). Cell lysates were subjected to immunoblotting with antibodies against IκBα. β-actin was used as a loading control in the corresponding lanes. (**B**) Signal intensity of IκBα bands were normalized by the signal intensity of β-actin band. High D-glucose (HG) increased the IκBα protein levels in the cells, an observation previously described by US (1). Treatment with terreic acid restored the IκBα levels similar to the control cells (NG-grown cells). *N* = 4 each group, Asterisks represent *p* ≤ 0.05. (**C**) BTK-inhibitor terreic acid (30 µM final concentration) decreased the elevated phosphorylation on Ser661 site of PKCβ in mouse primary endothelial cells grown in high D-glucose (HG) condition for 3 days. Cells were exposed to either normal glucose (NG, 5 mM D-glucose), high glucose (HG, 25 mM D-glucose), or high glucose with terreic acid (HG-TA, 25 mM D-glucose + 30 µM terreic acid). The lower part of the membrane was probed with antibody against β-actin (loading control). Results are presented as the ratio of PKC-Ser661 normalized by the signal intensity of β-actin in corresponding lanes. *N* = 3 samples for each condition. Asterisks denote statistically significant difference of *p *≤ 0.05.

Previously, we have shown that high glucose conditions impair the canonical NF-κB signaling resulting in increased steady state levels of IκBα (1) which was related to increased phosphorylation of PKCβ-Ser661. To test whether BLNK/BTK axis might be upstream of this pathway, we treated high D-glucose grown primary mouse endothelial cells (MVECsk) with terreic acid (TA, 20 and 30 µM), a selective inhibitor of BTK. Compared to the control cells grown in high L-glucose (normal D-glucose), cells grown in high D-glucose had increased IκB*α* protein (46.7% increase from control), an observation consistent with decreased NF-κB activity as we showed previously (Figures 8A,B) (1). Treatment with terreic acid significantly decreased accumulation of IκBα suggesting a net increase in NF-κB activity.

We further tested the effect of inhibiting BLNK/BTK signaling in primary mouse endothelial cells on PKC activation by phosphorylation. PKC phosphorylation was increased in high D-glucose conditions and treatment with terreic acid decreased this phosphorylation (Figures 8C,D). These results suggest that a BLNK/BTK mediated signaling pathway plays a role upstream of PKCβ in high glucose-related NF-κB signaling in endothelial cells.

## Discussion

In this study, using an antibody array for proteins and bioinformatics tools, we have identified BLNK and BTK proteins as novel upstream signaling components of hyperglycemic-ischemic NF-κB responses in endothelial cells. Phosphorylation and dephosphorylation are important biochemical modifications in propagation of NF-κB signaling. Functional NF-κB pathway is required for recovery from ischemic conditions (16), but prolonged high glucose and ischemic conditions impair the canonical pathway of the NF-κB signaling (1). We used an array of about 215 specific antibodies representing 100 distinct phospho-protein sites related to the NF-κB signaling. We compared the changes in phosphorylation of these proteins in the lysates from endothelial cells that were grown in normal D-glucose or high D-glucose under normoxic or ischemic conditions. Our results revealed a novel pathway of endothelial cells under hyperglycemic-ischemic conditions that utilizes proteins involved in B cell receptor signaling. Moreover, our analysis suggests that in accordance with our published results, hyperglycemic conditions alter the phosphorylation of various proteins participating in the NF-κB signaling to downregulate the canonical pathway (Figure 4). BLNK is a scaffold protein that can nucleate a large protein complex of several proteins including Tec family kinases of which BTK is a member (45). Although originally described in B-cell receptor signaling, BLNK/BTK pathway also functions in non-hematopoietic cells (39, 40). Thus, modulation of IκBα by knocking down BLNK expression and by a selective inhibitor of BTK suggest an important role of this pathway in hyperglycemia related inflammatory response. Further studies will elucidate the contribution of other proteins that function upstream of BLNK in impairment of the canonical NF-κB signaling in endothelial cells chronically exposed to high glucose.

Both high glucose concentration and ischemia elicit stress response in cells through activation of the NF-κB pathway. Accordingly, a set of common protein sites had increased phosphorylation in both cells grown in normal or high D-glucose when exposed to ischemic condition. Similarly, a set of protein sites had decreased phosphorylation under ischemic condition with normal or high D-glucose. However, we also identified protein phosphorylation sites that were uniquely modulated in cells exposed to normal glucose with ischemia or to high glucose with ischemia.

Based on our findings, we have constructed a schematic of putative signaling pathways that depicts interactions of components operating in balancing the canonical and non-canonical pathways of the NF-κB-mediated transcription activation (Figure 4). Here we discuss the phosphorylation events that were upregulated or downregulated specifically in HGI samples. We show that the upregulated phosphoproteins promote the non-canonical NF-κB pathway whereas the downregulated phosphoproteins limit the activation of the canonical NF-κB pathway. These changes in turn may have a net adverse effect of attenuating the canonical NF-κB pathway in hyperglycemic ischemic conditions.

### Upregulated phosphorylation sites

#### BLNK (p-Tyr96 and p-Tyr84)

B cell linker protein (BLNK) serves a scaffolding function to coordinate second messenger generation and signal transduction upon activation of B cell receptor (BCR). BLNK does not have an intrinsic enzyme activity; instead, it functions as a scaffold protein to assemble multiple proteins including kinases and PLCβ. In mouse, BLNK^−/−^ B cells show intact activation of AKT but impaired activation of the canonical nuclear factor NF-κB due to a failure to degrade IκBα protein (46). In accordance, we observed that shRNA-mediated knockdown of BLNK in mouse endothelial cells increased IκBα levels suggesting an impaired canonical NF-κB pathway (Figure 6). In this pathway, phospholipase C (PLC)-gamma2 has also been demonstrated to be essential for NF-κB activation. BLNK is required for PLCγ2 phosphorylation and Ca^2+^ influx through BCR activation (47). Peptide containing phosphorylated-Tyr96 residue specifically bind to BTK that leads to Ca^2+^ influx and PLCγ binding and phosphorylation upon BCR activation (29). Thus, the presence of BLNK in cultured primary endothelial cells (passage 4–7), that are devoid of B cells, suggests the presence of a B cell like signaling pathway. Consistent with our previous report of decreased canonical NF-κB pathway activation, we observed decreased PLCγ phosphorylation, whereas increased AKT1 phosphorylation leads to sustained or increased non-canonical activation of NF-κB pathway.

We also observed expression of BTK in both mouse and human primary endothelial cells from different tissue sources. We verified the expression of BTK transcripts in endothelial cells using QPCR based method. In addition, inhibition of BTK activity led to change in IκBα levels. These findings further suggest a functional role of BLNK/BTK pathway in inflammatory response in endothelial cells under hyperglycemic conditions. Interestingly, knocking down of BLNK or inhibition of BTK, both resulted in activated NF-κB as measured by changes in IκBa levels. Since the outcome of signaling through both BLNK and BTK depends on phosphorylation status of their different amino acid residues, future experiments will elucidate how these post-translational modifications relate to hyperglycemic and ischemic conditions.

#### AKT1 (p-Tyr474)

Akt1 is a key serine/threonine-protein kinase that regulates a number of cellular processes including cell survival, proliferation, metabolism, and angiogenesis by phosphorylating a number of protein substrate. However, the kinase activity of AKT1 itself is dependent on the phosphorylation of three specific sites (Thr308, Ser473 and Tyr474). Phosphorylation of Tyr474 residue, which was increased in our HGI samples, is the major determinant of AKT1 activity (24). The upstream signaling events leading to membrane localization and phosphorylation of AKT1 are coordinated by BTK, a non-receptor tyrosine kinase that acts as a scaffold protein to nucleate several signaling proteins. Active AKT1 positively affects a role in NF-κB-dependent gene transcription. Overexpression of constitutively active AKT1 increases non-canonical NF-κB activity by increasing IKK*α* activity that in turn results in increased production of p52 subunit of NF-κB (48). Our finding of increased Tyr474 phosphorylation of AKT1 in HGI cell lysates is consistent with our previous finding of increased non-canonical NF-κB activity in hyperglycemia both *in vitro* in HUVEC and *in vivo* in DM (1).

#### Ras-GRF1 (p-Ser916)

Ras-GRF1 (Ras-specific guanine nucleotide-releasing factor 1), also known as CDC25, is an exchange factor that promotes exchange of Ras-bound GDP by GTP. Ras-GRF1 is phosphorylated in an LCK-Syk dependent manner (49). Ras-GRF1 is linked to H-Ras and participates in activation pathway of ERK and canonical pathway of the NF-κB through CARD9-BCL10-MALT1 complex (25, 26).

#### HDAC1 (p-Ser916)

Histone deacetylase 1 (HDAC1) is a class I histone deacetylase that exists in multi protein complexes and modulates transcription through its enzyme activity. The phosphorylation of Ser421 residue of HDAC1 promotes its enzyme activity and complex formation (50). HDAC1 is thought to be associated with NF-κB activation since treatment of RAW264.7 cells with lovastatin inhibited IκBα phosphorylation as well as HDAC1 expression (27). Thus, HDAC1 in likely involved in down-regulating the canonical NF-κB pathway.

#### IKK gamma (p-Ser85)

IKKγ, also known as NEMO (NF-κB Essential Modulator), is a regulatory subunit of the cytoplasmic IKK complex. The IKK complex undergoes multiple post-translational modifications including phosphorylation. IKKγ is phosphorylated by PKCα (51). Phosphorylation of Ser85 of IKKγ enhances the kinase activity of IKKβ that results in increased phosphorylation of IκB. However, Ser85 alone is not sufficient for this activation; a combination of Ser85 and Ser141 is required for this enhancement. Moreover, IKKγ-Ser85 does not play a role in all NF-κB activation pathways (30). Our array did not contain antibody to Ser141, limiting a conclusion based only on IKKγ-Ser85 phosphorylation.

#### NF-κB-p100 (p-Ser872)

The p100 protein is the precursor of the p52 NF-κB subunit that undergoes phosphorylation-dependent inducible processing. Phosphorylation results in ubiquitination and proteosomal processing of p100 to generate p52 subunit that is translocated to the nucleus (52). The Ser872 site of p100 is phosphorylated by IKKα and is a requirement for ubiquitination and proteosomal processing of p100 protein (28). We have previously shown that under hyperglycemic conditions, the non-canonical NF-κB pathway preferentially remains active in HUVEC (1). The current finding of increased phosphorylation of p100 NF-κB subunit in HGI samples is consistent with increased role of the non-canonical NF-κB pathway.

#### P38 MAPK (p-Thr180)

The p38 MAPK is involved in expression of proinflammatory cytokines (53). Several environmental factors specifically induce p38 MAPK in signal specific manner by dual phosphorylation of Thr180 and Tyr182 residues (21). The NF-κB activity is affected by p38 MAPK since inhibitors of p38 result in diminished expression of NF-κB dependent genes (54) likely through post translational modification of the NF-κB subunits by phosphorylation or acetylation (22, 23) or by promoting transcription initiation complex on the target genes (55).

### Downregulated phosphorylation sites

#### LCK (p-Tyr505)

Lymphocyte cell kinase (LCK) is a Src-family kinase expressed predominantly in T cells and plays a key role in T-cell receptor (TCR) signaling pathway. However, LCK is expressed in endothelial cells, where its inhibition promoted endothelial proliferation and blocked apoptosis (56). Both structural and biochemical studies show that phosphorylation of the C-terminal 505 tyrosine residue (Y505) of LCK confers a closed molecular conformation, leading to inactivation of the kinase domain (57). In contrast, deletion or mutation of Y505 in LCK results in a constitutively active enzyme (58). Thus, decreased Tyr505 phosphorylation in high D-glucose plus ischemia would suggest an increase enzymatic activity of LCK (59).

#### I kappa B alpha (p-Ser32, Ser36)

Under the normal conditions, I kappa B alpha (IκBα) is an unstable protein that binds and retains the NF-κB transcription factor in the cytoplasm of the cells. Upon stimulation by appropriate signal, IκBα is rapidly degraded allowing the NF-κB transcription factor to migrate to the nucleus. The degradation of IκBα is dependent on phosphorylation at its Serine 32 and Serine 36 predominantly by IKKalpha/beta kinases. In addition to IKK complex, other kinases also phosphorylate IκBα on S32 and S36 (35). For the canonical pathway mediated NF-κB activation, phosphorylation of these two amino acid residues, S32 and S36, is an obligatory step. Thus, decreased phosphorylation of these residues on IκBα under our experimental conditions, i.e., high glucose with ischemia, suggests a decreased signaling through the canonical pathway. This finding is consistent with our published results that prolonged exposure of HUVEC to high D-glucose attenuated degradation of IκBα (1).

#### NF-κB-p65 (p-Thr435)

The inducing or repressing transcription activity of the p65 (RelA) subunit of the NF-κB transcription factor is modulated by phosphorylation. The p65 subunit is phosphorylated at multiple sites. TNF-α, a potent activator of the canonical NF-κB signaling pathway, induces phosphorylation of Thr435 residue of the p65 in the transcription activation domain. This phosphorylation increases occupancy by p65 in a highly promoter-specific manner (36) to increase the expression of proinflammatory chemokine. Thus, a decreased phosphorylation of p65-T435 would effectively decrease the induction of certain genes affected by the canonical NF-κB pathway.

#### PKCtheta (p-Ser676) and PKCzeta (p-Thr410)

Protein kinase C-theta (PKCθ) and PKCzeta are members of atypical serine/threonine kinase PKC family that plays a role in NF-κB activation in T lymphocytes (31) for adequate immune response. The function of the PKC family members is regulated by phosphorylation at several different residues. PKC are phosphorylated on at least 3 sites after T cell receptor stimulation (60). The p-Ser676 site in PKCθ is in the turn-motif of the enzyme that is autophosphorylated (61). Thus, a decrease in phosphorylation at this site might reflect the decreased enzyme activity. Overexpression of PKCζ specifically increases IKKβ activity without affecting IKKα. The Thr410 residue of PKCζ is located in its activation domain and its phosphorylation is critical for PKCζ activity (62). Since both PKCθ and PKCζ are involved in induction of NF-κB by phosphorylating IKK (32). In addition, PKCζ can directly phosphorylated the RelA subunit of NF-κB transcription factor (34). Taken together, decreased enzyme activity of PKCθ and PKCζ would result in a decline in canonical NF-κB activation.

#### PKR (p-Thr446 and p-451)

PKR is a double stranded RNA (dsRNA)-activated serine/threonine protein kinase that potentiates the activation of NF-κB by phosphorylating IκB (33). PKR is autophosphorylated at multiple residues including Thr446 that is associated with stabilization of homodimeric form and increased catalytic activity (63). Decreased phosphorylation of Thr446 is consistent with concomitant decrease in canonical NF-κB activity.

#### SAPK/JNK (p-Thr183)

Stress activated protein kinase/c-Jun-N-terminal kinase 1 (SAPK/JNK) is activated by phosphorylation at positions Thr183 and Tyr185 (64). JNK may participate in inflammatory process by promoting NF-κB action on specific promoters (65).

Thus, we have demonstrated that hyperglycemia and ischemia, either alone or in combination, cause profound changes in the phosphorylation states of the components of the key inflammatory pathway- the NF-κB. Importantly, we have identified BLNK and BTK as novel upstream components of the NF-κB inflammatory pathway in hyperglycemic-ischemic condition. In the future, it will be important to study how BLNK/BTK are activated by diabetes-related hyperglycemia.

## Data Availability

The raw data supporting the conclusions of this article will be made available by the authors, without undue reservation.
